# Flutamide induces alterations in the cell-cell junction ultrastructure and reduces the expression of Cx43 at the blood-testis barrier with no disturbance in the rat seminiferous tubule morphology

**DOI:** 10.1186/s12958-016-0144-2

**Published:** 2016-03-31

**Authors:** Katarzyna Chojnacka, Anna Hejmej, Marta Zarzycka, Waclaw Tworzydlo, Szczepan Bilinski, Laura Pardyak, Alicja Kaminska, Barbara Bilinska

**Affiliations:** Department of Endocrinology, Institute of Zoology, Jagiellonian University, Krakow, Poland; Department of Developmental Biology and Morphology of Invertebrates, Institute of Zoology, Jagiellonian University, Krakow, Poland

**Keywords:** Flutamide, Cx43, Basal ectoplasmic specialization, Testis, Rat, Ultrastructure

## Abstract

**Background:**

Present study was designed to establish a causal connection between changes in the cell-cell junction protein expression at the blood-testis barrier and alterations in the adult rat testis histology following an anti-androgen flutamide exposure. Particular emphasis was placed on the basal ectoplasmic specialization (ES) in the seminiferous epithelium and expression of gap junction protein, connexin 43 (Cx43).

**Methods:**

Flutamide (50 mg/kg body weight) was administered to male rats daily from 82 to 88 postnatal day. Testes from 90-day-old control and flutamide-exposed rats were used for all analyses. Testis morphology was analyzed using light and electron microscopy. Gene and protein expressions were analyzed by real-time RT-PCR and Western blotting, respectively, protein distribution by immunohistochemistry, and steroid hormone concentrations by radioimmunoassay.

**Results:**

Seminiferous epithelium of both groups of rats displayed normal histology without any loss of germ cells. In accord, no difference in the apoptosis and proliferation level was found between control and treated groups. As shown by examination of semi-thin and ultrathin sections, cell surface occupied by the basal ES connecting neighboring Sertoli cells and the number of gap and tight junctions coexisting with the basal ES were apparently reduced in flutamide-treated rats. Moreover, the appearance of unconventional circular ES suggests enhanced internalization and degradation of the basal ES. These changes were accompanied by decreased Cx43 and ZO-1 expression (*p* < 0.01) and a loss of linear distribution of these proteins at the region of the blood-testis barrier. On the other hand, Cx43 expression in the interstitial tissue of flutamide-treated rats increased (*p* < 0.01), which could be associated with Leydig cell hypertrophy. Concomitantly, both intratesticular testosterone and estradiol concentrations were elevated (*p* < 0.01), but testosterone to estradiol ratio decreased significantly (*p* < 0.05) in flutamide-treated rats compared to the controls.

**Conclusions:**

Short-term treatment with flutamide applied to adult rats exerts its primary effect on the basal ES, coexisting junctional complexes and their constituent proteins Cx43 and ZO-1, without any apparent morphological alterations in the seminiferous epithelium. In the interstitial compartment, however, short-term exposure leads to both histological and functional changes of the Leydig cells.

## Background

In the testis, Sertoli cells serve as supporting cells and reside as a basal epithelial lining within the seminiferous epithelium. They create a specialized microenvironment to support the germ cell development especially through the formation of the blood-testis barrier (BTB). The BTB is different from most other tissue barriers; its structural components include not only tight junctions (TJs), but also gap junctions (GJs) and atypical adherens junctional complexes - ectoplasmic specialization (ES) [1, 2]. The basal ES is found between adjacent Sertoli cells and their projections at the BTB, while the apical ES is limited to the interface between the Sertoli cell and elongating spermatids [3, 4].

Several lines of evidence indicate that not only TJ but also GJ are required for adequate functioning of the testis. In adult male, connexin 43 (Cx43), the most abundant testicular GJ protein is predominantly localized at the region of BTB and among Leydig cells within the interstitial tissue [5, 6]. Studies on Sertoli cell-specific deletion of Cx43 revealed that this connexin is an absolute requirement for the Sertoli cell development and initiation of spermatogenesis [7, 8]. Several studies on human, rodents [9, 10], and non-rodent species such as stallion, European bison, and pig [11–13] have documented alterations in Cx43 expression in seminiferous tubules as associated with impaired spermatogenesis.

Testosterone is essential for male fertility, mediating its biological effects through the androgen receptor (AR) present in somatic Leydig, peritubular, and Sertoli cells. It has been shown that testosterone is a direct regulator of BTB function and dynamics [14]. Strong evidence for an important role of testosterone acting *via* AR for the maintenance of BTB integrity was provided by the studies of Sertoli-cell specific AR knockout (SCARKO) mice [15]. Since unbalanced ratio of the active androgens can lead to various structural and functional abnormalities within the testes, analyses of the effects of environmental or chemical compounds antagonizing physiological androgen action are of potential importance [16, 17].

Flutamide, a pharmaceutical non-steroidal anti-androgen discovered as a treatment for androgen-dependent prostate cancer, acts as a competitive antagonist that inhibits the effects of androgens through AR blockade [18]. Thus in experimental studies, flutamide is often used to study the role of androgen receptor signaling in physiological and pathological processes. Taking into account our previous studies showing affected spermatogenesis in boars treated with flutamide neonatally [13, 19] and recent observations showing altered immunoexpression of Cx43 at the BTB level after flutamide exposure of adult rat [20] and in primary rat Sertoli cells *in vitro* [21], we hypothesize that limited exposure to flutamide may have a direct impact on the *Cx43* gene expression in the testis, which may precede an impact on the tissue histology. To assess this we applied short, seven-day-treatment with flutamide and examined rat seminiferous tubule morphology and Cx43 expression at the mRNA and protein level. The effects of flutamide on apoptosis and proliferation of testicular cells were also investigated. To get a deeper insight into the action of flutamide on the morphology of the BTB, we studied the organization of the basal ES and intercellular junctions residing in this region at the ultrastructural level. Since within the BTB, the tight and gap junctions are located next to each other, we finally examined the expression of the tight junction-associated protein, zonula occludens-1 (ZO-1). Elucidation of a causal connection between changes in the Cx43 and ZO-1 expression within the tubule and alterations in the seminiferous epithelium morphology may be of importance for understanding and predicting fertility disorders induced by anti-androgens.

## Methods

### Animals and experimental design

Eighty-two-day old male Wistar rats (*Rattus norvegicus*) (~300 g b. w.) originating from five litters were allotted into experimental (*n* = 6) and control (*n* = 6) groups. Rats were kept in the animal house of the Institute of Zoology, Jagiellonian University in Krakow in a controlled environment with a temperature of 22 ± 2 °C at 55 ± 5 % humidity and a 12-h light/dark cycle. Standard pellet food (Motycz, Poland) and tap water were available *ad libitum*. Experimental rats were injected subcutaneously with 50 mg⁄ kg b.w. of flutamide (2-methyl-N-[4-nitro-3-(trifluoromethyl)-phenyl]propamide; Sigma–Aldrich, St. Louis, MO, USA, suspended in corn oil) daily for seven consecutive days (82 – 88 day of postnatal life). Rats from the control group were given corn oil only. Animals were maintained until 90 days of age when the testes were removed.

According to our hypothesis, to discriminate a primary effect of androgen signaling disruption by flutamide, it was important to select a dose, frequency, and time of flutamide treatment which was high enough to induce changes in the expression of intercellular junction proteins within seminiferous epithelium without causing alterations in testicular cell morphology and producing a toxic effect in Sertoli cells *in vitro*. Thus treatment protocol was based on the literature data and our previous studies [20–22].

The use of animals reported herein was approved by the First Local Ethical Committee on Animal Testing at the Jagiellonian University in Krakow (approval No. 116/2012).

### Tissue preparation

Both testes of each individual of control and flutamide-treated rats were surgically removed and cut into small fragments. For histology and immunohistochemistry, tissue samples were fixed in 4 % formaldehyde freshly prepared from paraformaldehyde and embedded in paraplast. Other tissue fragments were immediately frozen in a liquid nitrogen and stored at −80 °C for RNA isolation, protein extraction, and determination of hormone levels in testes homogenates.

### Morphology

For routine histology, hematoxylin-eosin (H-E) staining was performed. The sections were examined under Nikon Eclipse Ni-U microscope (Nikon, Tokyo, Japan). A tubus setting of 1.25, a × 10 ocular, and a × 10 objective were used for the measurements. Detailed morphologic analysis was performed with the use of NIS-Elements software (Nikon, Tokyo, Japan), as previously described [23]. Diameter of 50 seminiferous tubules profiles that were round or nearly round were measured, and a mean was determined for control and treatment groups. The area of the interstitium occupied by Leydig cells was determined in 40 random fields of vision (which corresponds to 17.7 mm^2^) for each animal from control and treated groups. Then, Leydig cells were counted, and the mean number of the cells per 1 mm^2^ of the interstitial tissue was determined for each section.

### Electron microscopy (EM) studies

The fixation procedure described below was based on the protocols proposed by Russell [24, 25]. The modification developed in our labs had important advantages: it improved the quality of fixation and enhanced the contrast of plasma membranes, allowing observation of ectoplasmic specializations even at the level of light microscopy. Briefly, dissected testes of control and flutamide-treated rats were immersed in ice-cold pre-fixative containing 2 % formaldehyde and 2.5 % glutaraldehyde in 0.1 M phosphate buffer, pH 7.3. Then individual seminiferous tubules were isolated and fixed in the same fixative for 2 h at 4 °C. The tubules were then rinsed and post-fixed in a mixture of 2 % osmium tetroxide and 0.8 % potassium ferrocyanide in the same buffer for 30 min at 4 °C. The material was embedded in Glycid Ether 100 resin (Serva, Heidelberg, Germany). Semi-thin sections (0.7 μm thick) were stained with 1 % methylene blue and examined under a Leica DMR (Wetzlar, Germany) microscope. Prior to embedding small (3–5 mm) pieces of seminiferous tubules were carefully oriented in the mold to obtain accurate cross sections of the tubules. Ultrathin sections (80 nm thick) were contrasted with uranyl acetate and lead citrate and analyzed with a JEOL 2100 HT (Japan) TEM.

**Fig. 1:**
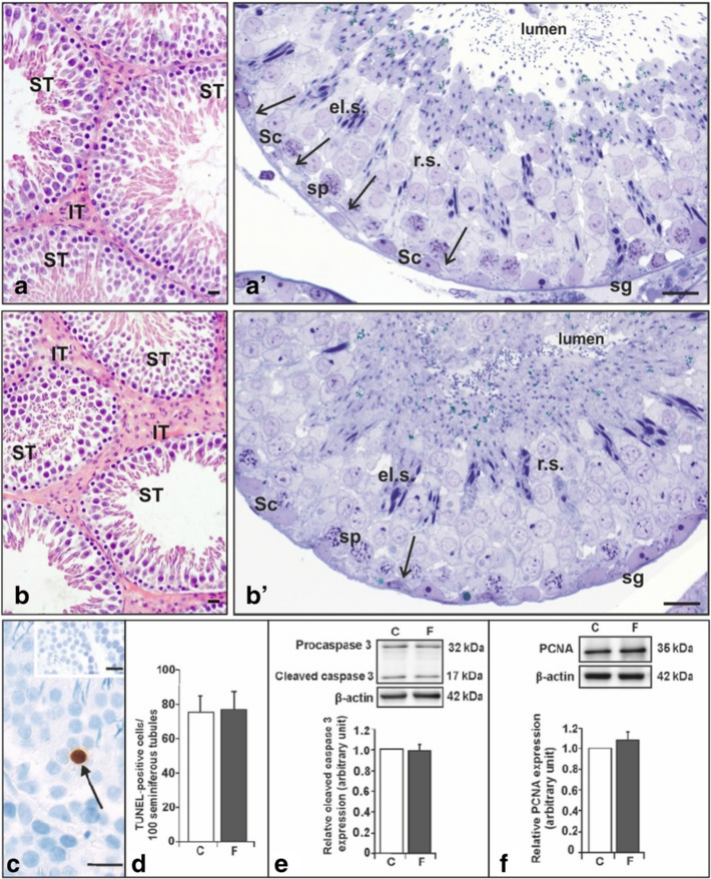
**a**-**f** Testis morphology (**a**-**b**), apoptosis (**c**-**d**) and proliferation (**e**-**f**) in control and flutamide-treated rats. **a**-**b** Morphology of the testis. Representative section of control (**a**) and flutamide-treated (**b**) rats. H-E staining. Scale bars represent 15 μm. Note full spermatogenesis in seminiferous tubules (ST) of both control and flutamide-treated testes (**a**-**b**) and enlarged interstitial tissue (IT) after flutamide exposure (**b**). Representative semi-thin section of control (*a’*) and flutamide-treated rats (*b’*). Scale bars represent 15 μm. Both cross-sections (*a’*, *b’*) illustrate different cell types present during spermatogenesis. From the basal membrane to the lumen: spermatogonia (sg), spermatocytes (sp), round spermatids (r.s) and elongated spermatids (e.s) are visible. Sertoli cell (Sc) nucleus is situated close to the basal membrane, while Sertoli cell cytoplasm extends towards the lumen being in close contact with the other cell types. Note any loss of germ cells within the seminiferous epithelium (*a’*, *b’*). Abundant distribution of ectoplasmic specializations (*arrows*) close to the basal membrane of control testis (*a’*). Note the reduction of the cell surface occupied by the basal ectoplasmic specializations connecting adjoined Sertoli cells (arrow) following flutamide treatment (*b’*). C-F. TUNEL staining, caspase 3 protein, and proliferating nuclear antigen (PCNA) expression in testes of control and flutamide-treated rats. Representative TUNEL staining (*arrow*, **c**). Scale bars represent 15 μm. Immunoreactive cells were absent in negative control sections (**c**, *top*). Quantitative analysis of TUNEL-positive cells (**d**). Representative immunoblots for caspase 3 (**e**, *top*) and PCNA (**f**, *top*). The band at 32 kDa represents inactive proenzyme (procaspase 3) and the band at 17 kDa corresponds to active form (cleaved caspase 3). Actin was used as a loading control, and each set of shown actin immunoblots corresponds to the target protein that was investigated within a given panel. The relative level of cleaved caspase 3 (**e**, *bottom*) and PCNA (**f**, *bottom*) protein normalized against its corresponding *β*-actin. Data obtained from three separate analyses is expressed as mean ± SD. Control (*n* = 6) and flutamide (*n* = 6) group

### TUNEL assay

The presence of apoptosis-related DNA strand breaks in testicular cells was evaluated by TUNEL assay using the In situ Cell Death Detection kit, POD (Roche, Mannheim, Germany) according to the manufacturer’s instructions. Apoptotic cells were observed under bright field optics in Leica DMR microscope (Leica Microsystems, Wetzlar, Germany) using a 40 × objective, and scored by an observer blinded to the treatment groups. For each testicular cross section, all cross-sectional tubular profiles (∼100) were counted and the number of TUNEL-positive cells noted for each cross-sectioned tubule.

### Immunohistochemistry: qualitative and quantitative evaluation

To optimize immunohistochemical staining the sections were immersed in 10 mM citrate buffer (pH 6.0) and heated in a microwave oven (2 × 5 min, 700 W). Thereafter, sections were immersed sequentially in H_2_O_2_ (3 %; v/v) for 10 min and normal goat serum (5 %; v/v) for 30 min which were used as blocking solutions. After overnight incubation with rabbit polyclonal antibody against Cx43 (1: 2000; Sigma-Aldrich, St Louis, MO, USA) or mouse monoclonal antibody against ZO-1 (1 : 500; Invitrogen) at 4 °C, biotinylated antibodies (goat anti-rabbit and anti-mouse IgGs; 1: 400; Vector, Burlingame CA, USA) and avidin-biotinylated horseradish peroxidase complex (ABC/HRP; 1:100; Dako, Glostrup, Denmark) were applied in succession. Bound antibody was visualized with 3,3’-diaminobenzidine (0.05 %; v/v; Sigma-Aldrich) as a chromogenic substrate. Control sections included omission of primary antibody and substitution by irrelevant IgG. The whole procedure was described in detail elsewhere [13, 19]. Experiments were repeated three times. The sections were examined with a Leica DMR microscope (Wetzlar, Germany). To evaluate the intensity of immunohistochemical reaction quantitatively, digital images were obtained and analyzed using a public domain ImageJ software (National Institutes of Health, Bethesda, Maryland, USA). The intensity of the immunostaining was calculated using the formula described by Smolen [26] and expressed as relative optical density (ROD) of diaminobenzidine brown reaction products. A total number of 20 testis sections (*n* = 10 per group) were subjected to image analysis and results of 10 separate measurements were expressed as mean ± SD.

### Western blot analysis

Lysates were obtained by sample homogenization and sonication with a cold Tris/EDTA buffer (50 mM Tris, 1 mM EDTA, pH 7.5), supplemented with a broad-spectrum protease inhibitors (Sigma-Aldrich). The protein concentration was estimated by the Bio-Rad DC Protein Assay Kit with BSA as a standard (Bio-Rad Labs, GmbH, München, Germany). Equal amounts of protein were resolved by SDS-PAGE under reducing conditions, transferred to polyvinylidene difluoride membranes (Merck Millipore, Darmstadt, Germany) and analyzed by Western blotting with: rabbit polyclonal antibodies against Cx43 (1 : 12 000; Sigma-Aldrich) or caspase 3 (1 : 1000; Cell Signaling Technology, Beverly, MA, USA), or mouse monoclonal antibodies against ZO-1 (1 : 500; Invitrogen) or PCNA (1 : 500; Merck Millipore), as previously reported [19]. The presence of the primary antibody was revealed with horseradish peroxidase-conjugated secondary antibodies diluted 1:3000 (Vector Lab., Burlingame, CA, USA) and visualized with an enhanced chemiluminescence detection system as previously described [20]. Actin served as a loading control.

To obtain quantitative results the bands (representing each data point) were densitometrically scanned using the public domain ImageJ software (National Institute of Health, Bethesda, MD, USA) and the data obtained for each protein were normalized against its corresponding β-actin. Protein level within the control group was arbitrarily set as 1.

### RNA Isolation, Reverse Transcription

Total RNA was extracted from testes using TRIzol® reagent (Life Technologies, Gaithersburg, MD, USA) according to the manufacturer’s instructions. To remove contaminating DNA and DNase from RNA preparations, the RNA samples were incubated with reagents from the TURBO DNA-free™ Kit (Ambion, Austin, TX). The yield and quality of the RNA were assessed using a NanoDrop ND2000 Spectrophotometer (Thermo Scientific, Wilmington, DE, USA) and by electrophoresis. Total cDNA was prepared using High-Capacity cDNA Reverse Transcription Kit (Applied Biosystems, Carlsbad, CA, USA) according to the manufacturer’s instructions.

### Real-Time Quantitative RT-PCR

Real-time RT-PCR was performed using the StepOne Real-Time PCR system (Applied Biosystems) and optimized standard conditions as described previously [20]. The mRNA expression levels of the Cx43 and ZO-1 were quantified in each sample using TaqMan Gene Expression Assays (Applied Biosystems) as follows: for Cx43 assay ID, Rn01433957_m1; for ZO-1 assay ID, Rn02116071_s1; GAPDH levels were determined as an endogenous control assay (Applied Biosystems, assay ID, Rn01775763_g1). Control reactions either without the RNA template or without the reverse transcriptase enzyme were performed. Relative quantification (RQ) was obtained using the 2 − ΔΔCt method, adjusting the Cx43 and ZO-1 mRNAs expression to the expression of GAPDH mRNA and taking the adjusted expression in the control group as reference (RQ = 1) [27]. Three independent experiments were performed, each in triplicate with tissues prepared from different animals. All PCR products stained with Midori Green Stain (Nippon Genetics Europe GmbH, Düren, Germany) were run on 2.5 % agarose gels. Images were captured using a Bio–Rad Gel Doc XR System (Bio–Rad Laboratories, Hercules, CA, USA).

### Radioimmunological analysis

Homogenized testicular tissues from control and flutamide-treated rats were used for radioimmunological determination of testosterone and estradiol levels as described previously [28]. Testosterone levels were assessed using [1,2,6,7-^3^H]-testosterone (The Radiochemical Centre, Nycomed Amersham, Buckinghamshire, England), specific activity 88.0 Ci/mmol, as a tracer and antibody raised in rabbit against testosterone-3-(O-carboxymethyl) oxime-bovine serum albumin (BSA), whereas estradiol concentrations were determined using [2,4,6,7-^3^H]-estradiol (The Radiochemical Centre, Nycomed Amersham), specific activity 140 Ci/mmol, as a tracer and an antibody raised in rabbit against 17β-estradiol-6-(O-carboxymethyl) oxime-BSA. The within-assay coefficients of variation were < 8 %. All samples were assayed in duplicates from at least three separate experiments.

### Statistical analysis

Each variable was tested by using the Shapiro-Wilk *W*-test for normality. Homogeneity of variance was assessed with Levene’s test. Since the distribution of the variables was normal and the values were homogeneous in variance, all statistical analyses were performed using one-way analysis of variance (ANOVA) followed by Tukey’s *post hoc* comparison test to determine which values differed significantly from controls. The analysis was made using Statistica software (StatSoft, Tulsa, OK, USA). Data were presented as mean ± SD. Data were considered statistically significant at *p* < 0.05.

## Results

### Effect of flutamide on testis morphology

Spermatogonia, spermatocytes and haploid germ cells at advanced stages of differentiation (round and elongated spermatids) were observed in testes of control and flutamide-treated rats (Fig. 1a, a’ , b, b’). Both Sertoli and germ cells displayed normal morphology without any loss of germ cells irrespective of the group of rats. As shown on semi-thin sections, numerous ESs were observed in the control (Fig. 1a’), whereas flutamide treatment led to apparent reduction of the cell surface occupied by the basal ES connecting neighboring Sertoli cells and/or their projections (Fig. 1b’ , for details see also the section below). On the other hand, the interstitial area containing Leydig cells was enlarged after flutamide exposure (Fig. 1b). To analyze the effect of flutamide exposure quantitatively, seminiferous tubule diameter and area occupied by Leydig cells were measured, and the number of Leydig cells per unit area was counted. Seminiferous tubule diameter did not change, whereas the area occupied by Leydig cells increased significantly in flutamide-treated rats compared to the control. The number of Leydig cells per unit area of interstitial tissue was almost 2-fold reduced after flutamide exposure (Table 1).

**Table 1:** Diameter of the seminiferous tubules, the area occupied by Leydig cells and the number of Leydig cells per unit area of interstitial tissue in control and flutamide-exposed rats

	Control	Flutamide-treated
Diameter of the seminiferous tubules (μm)	287.80 ± 32.09	295.11 ± 35.98
The area occupied by Leydig cells per 40 fields of vision (mm^2^)	3.42 ± 0.11	5.18 ± 0.28*
The number of Leydig cells per 1 mm^2^ of interstitial tissue	5984.87 ± 905.91	3275.30 ± 390.41**

Data are expressed as means ± SD. Significant differences from control values are denoted as ^*^ *p* < 0.05 and ^**^ *p* < 0.05 (*n* = 6 animals/each group)

### Effect of flutamide on the ultrastructure of the basal ES

**Fig. 2:**
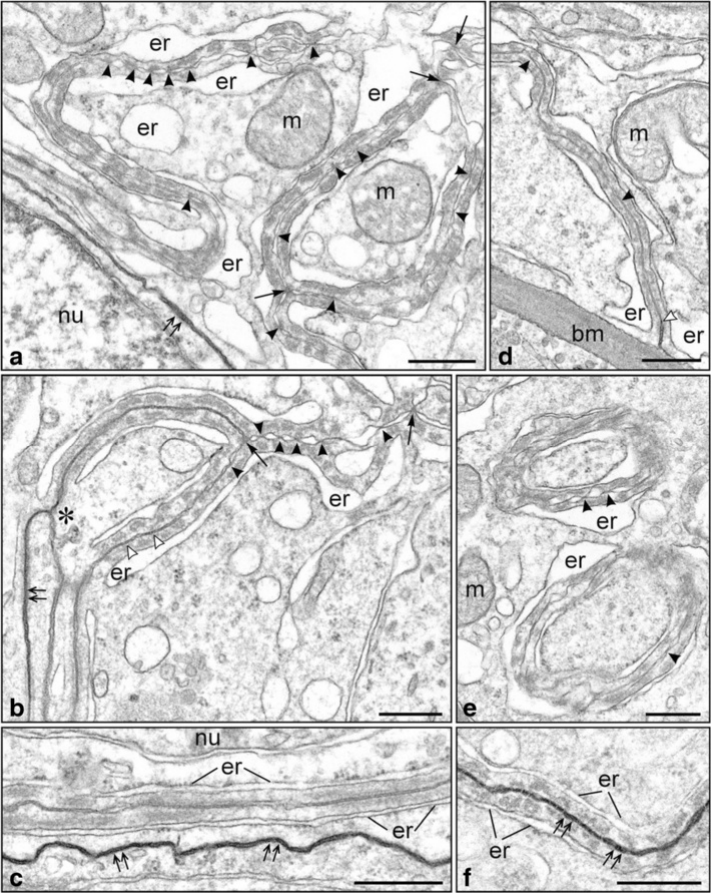
**a**-**f** Ultrastructure of basal ectoplasmic specialization in control (**a**-**c**) and flutamide-treated (**d**-**f**) rats. **a**-**c** Basal ectoplasmic specialization connecting adjoined Sertoli cells in control rats. Note that electron-dense tracer penetrates only between non-specialized membranes (*double* *arrows* in **a**, **b**, and **c**) and is stopped at the basal limit of ectoplasmic specialization (asterisk in **b**). Tangential section through the junctional complex (sandwiched between endoplasmic reticulum cisternae) located next to non-specialized membranes (**c**). Scale bars represent 0.5 μm. **d**-**f** Basal ectoplasmic specialization in flutamide-treated rat. Note loss of 3-membrane junctions (D); two circular ectoplasmic specializations in the cytoplasm of Sertoli cell (**e**); junctional complex (ectoplasmic specialization + tight junctions) penetrated by the tracer (*double arrows* in **f**). Scale bars represent 0.5 μm. Basement membrane – bm; endoplasmic reticulum – er; mitochondrium - m; Sertoli cell nucleus – nu. Arrows indicate 3-membrane junctions, open arrowheads – gap junctions, arrowheads – tight junctions

Analysis of serial semi-thin sections (compare Fig. 1a’ and b’) and ultra-thin sections showed that flutamide treatment led to the reduction of the cell surface occupied by the basal ES connecting adjoined Sertoli cells (compare Fig. 2a, b and d). Moreover, our analyses revealed that the number of 3-membrane junctions was much lower within the basal ES of flutamide-treated rats (Fig. 2d) when compared with the control ones (Fig. 2a, b). Above observations indicated that the number of cell projections interdigitated between adjacent Sertoli cells was distinctly reduced in flutamide-treated rats. This, in turn, coincided well with the observation of unconventional circular ES (Fig. 2e) observed in the neighborhood of the Sertoli cell surface of experimental animals. Such circular “segments” of the basal ES were most probably formed as a result of retraction of Sertoli cell projections and/or internalization of the junctional complexes by the Sertoli cell cytoplasm. It should be noted here that morphologically similar circular ES were never noted in the testes of control rats. The EM analyses also showed that the number of gap and tight junctions coexisting with the basal ES or located in their vicinity was reduced in treated rats, compared with the controls (compare Figs. 2d and a, b). Similar reduction was also observed in case of the circular ES that, as a rule, contained only single or few TJs (Fig. 2e).

In our fixation procedure potassium ferrocyanide (contained in the post-fixative) contrasted only “open” intercellular spaces, it did not penetrate between membranes sealed by tight junctions. Thus the procedure allowed also “visualization” of functional state of the basal ES, i.e. the permeability of the TJs located within the ES. In control rats the electron-dense tracer freely penetrated between non-specialized membranes (Fig. 2a-c) and was stopped at the basal limit (border) of the ES (Fig. 2b). In rats treated with flutamide, at least some junctional complexes (ES and coexisting TJs) were permeable and penetrated by the tracer (Fig. 2f).

### Effect of flutamide on apoptosis and proliferation in adult testis

To investigate the effect of androgen signaling disruption on apoptosis and proliferation in testes of flutamide-treated and control rats, *in situ* TUNEL assay and cleaved caspase 3 as an apoptotic marker, and proliferating cell nuclear antigen (PCNA) as a proliferation marker, were used, respectively (Fig. 1c-f).

TUNEL-labeled cells were rarely observed in the seminiferous epithelium of flutamide-treated and control rats (Fig. 1c). Apoptotic cells were mostly accounted for as spermatocytes. Nuclear staining pattern permitted to score apoptotic cell number, although no significant changes in TUNEL-positive cells frequency were observed after flutamide exposure compared with the control (Fig. 1d).

Using Western blot both procaspase 3 and cleaved caspase 3 were detected in testis homogenates of flutamide-treated and control rats (Fig. 1e). Densitometric analysis revealed no statistically significant differences between the level of cleaved caspase-3 in testes of flutamide-treated and control rats (Fig. 1e).

The expression of PCNA was also analyzed by Western blotting. Treatment with flutamide resulted in a slight, no statistically significant increase in the PCNA level (Fig. 1f).

### Effect of flutamide on the expression of mRNA and protein for Cx43 and its immunolocalization in adult testis

**Fig. 3:**
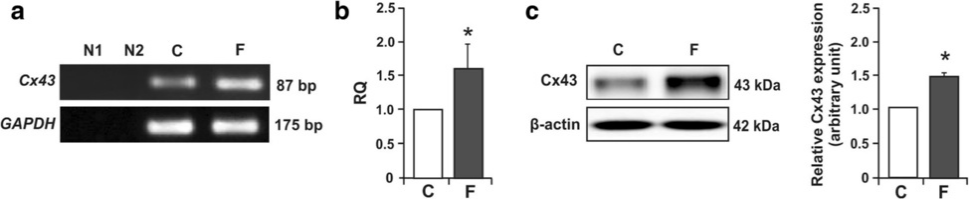
**a**-**c** Analyses of *Cx43* gene expression in testes of control and flutamide-treated rats. Representative RT-PCR (**a**), quantitative real-time PCR (**b**), and Western blot (**c**). As an internal control, the GAPDH mRNA level was measured in the samples. N1 – negative control without cDNA template. N2 – negative control without reverse-transcribed RNA. Relative quantification (RQ) is expressed as mean ± SD. The relative level of Cx43 protein normalized to β-actin which served as an internal protein loading control. Data obtained from three separated analysis are expressed as mean ± SD. Significant differences from control values are denoted as *p < 0.05. Control (*n* = 6) and flutamide (*n* = 6) group

To assess the effect of flutamide on Cx43 expression at mRNA and protein levels, RT-PCR and Western blot analysis were performed, respectively. Electrophoresis revealed PCR-amplified products of the predicted sizes; 87 bp for Cx43 and 175 bp for GAPDH in testes from flutamide-treated and control rats (Fig. 3a). Real-time RT-PCR revealed an up-regulation of Cx43 mRNA expression (*p* < 0.05) in flutamide-exposed rat testes (Fig. 3b). For negative controls, RT or cDNA was omitted from each reaction.

Western blot analysis revealed immunodetectable Cx43 as a single protein band near 43 kDa position of the gel, in testicular homogenates of flutamide-treated and control rats (Fig. 3c, left panel). In the testes of flutamide-treated rats, the expression level of Cx43 significantly increased (*p* < 0.05) compared with the control (Fig. 3c, right panel).

**Fig. 4:**
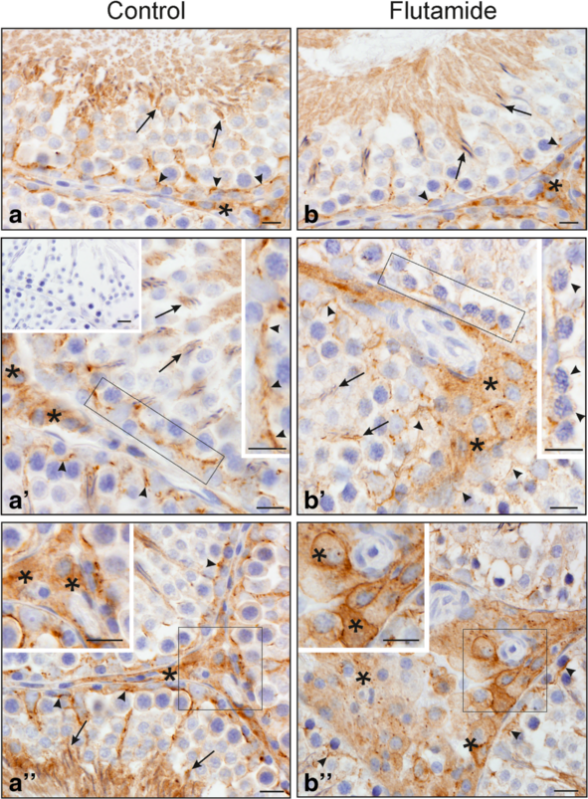
**a**-**b** Immunohistochemical localization of Cx43 in testes of control (**a**, *a’*, *a”*) and flutamide-treated (**b**, *b’*, *b”*) rats. Scale bars represent 15 μm. All sections were counterstained with Mayer’s haematoxylin. In control rats (**a**, *a’*, *a”*) Cx43 is distributed in linear array between adjacent Sertoli cells at the level of the BTB (*arrowheads*). For details, see the higher magnification in *a’*. Note also Cx43 signal between Sertoli cells and spermatocytes and at the Sertoli cell-elongated spermatid interface (arrows; **a**, *a’*). In the interstitium, a strong signal for Cx43 is localized to Leydig cells (asterisks; **a**, *a’*, *a”* and insert in *a”*). Following flutamide treatment (**b**, *b’*, *b”*), note apparent decrease in the Cx43 immunostaining at the BTB site (*arrowheads*). For details, see the higher magnification in B’. Similar signal intensity as in control testis is visible at the Sertoli cells-elongated spermatid interface (*arrows*; **b**, *b’*). Note very strong linear signal at cellular membrane between neighboring Leydig cells in the interstitium (*asterisks*; *b”* and insert in *b”*). Rectangles indicate the location of the higher magnification view. No signal was detected when anti-Cx43 antibody was substituted by normal goat serum (insert in *a’*)

As revealed by immunohistochemistry (see Fig. 4) flutamide led to the reduction of the Cx43 signal intensity at the base of the epithelium (Fig. 4b, b’), while a positive signal reminded almost unchanged at the apical compartment of the seminiferous epithelium compared with the control (Fig. 4a, a’). Within the interstitium, however, a very strong linear Cx43 signal on the plasma membrane of Leydig cells was found as the prevalent staining pattern (Fig. 4b”) compared with the control (Fig. 4a”). Thorough analysis (at higher magnification) revealed a loss of linear staining pattern at the region of the BTB (Fig. 4b’*versus *a’) what was confirmed by the optical density quantitative measurement. The signal was significantly reduced (*p* < 0.01) in flutamide-treated tissue (18.82 ± 1.15) compared with the control (39.61 ± 3.20). Further quantitative evaluation of the Cx43 signal also reflected the qualitative results. There was no statistically significant difference between the Cx43 signal at the Sertoli cell-elongated spermatid interface in flutamide-treated testis (32.38 ± 3.32) *versus* control one (38.15 ± 4.65), while in hypertrophic Leydig cells of flutamide-exposed animals the Cx43 signal was significantly higher (*p* < 0.01) than that of the control (43.05 ± 3.37 *versus* 25.58 ± 4.86).

### Effect of flutamide on ZO-1 immunolocalization and expression of mRNA and protein for ZO-1 in adult testis

**Fig. 5:**
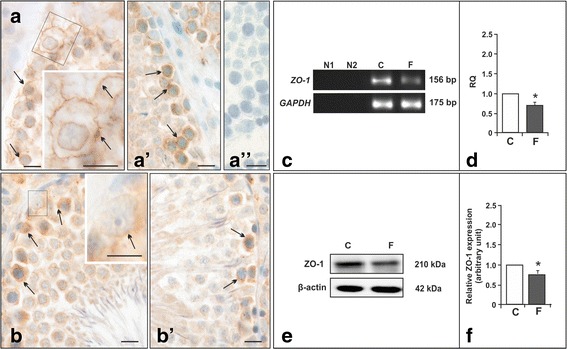
**a**-**e** Immunohistochemical localization of ZO-1, its mRNA, and protein expression in testes of control and flutamide-treated rats. **a**-**b** Immunohistochemical staining in testes of control (**a**, *a’*) and flutamide-treated rats (**b**, *b’*). Nomarski interference contrast. Scale bars represent 15 *μ*m. Arrows point to ZO-1 signal at the base of the tubule (**a**, *a’*, **b**, *b’*). Moderate to strong ZO-1 signal at the level of the BTB is visible as continuous wavy line between adjacent Sertoli cells (**a**, *a’*). Note ZO-1 signal which lines the cytoplasmic face of Sertoli cells (**a**, insert in **a**). Following flutamide treatment weak to moderate ZO-1 signal is visible mainly in the cell cytoplasm (**b**, *b’*). Rectangles indicate the location of the higher magnification views (**a**, **b**). No ZO-1 signal was detected when anti-ZO-1 antibody was substituted by normal goat serum (*a”*). Representative RT-PCR (C) and quantitative real-time PCR (D) analyses of ZO-1 mRNA expression in testes of control and flutamide-treated males. As an internal control, the GAPDH mRNA level was measured in the samples (**c**). Lane N1 – negative control without cDNA template, lane N2 – negative control without reverse-transcribed RNA. The relative amount of ZO-1 transcript levels (**d**). Relative quantification (RQ) is expressed as mean ± SD. Significant difference from control values is denoted as *p < 0.05. Control (*n* = 6) and flutamide (*n* = 6) group. Representative Western blot analysis of ZO-1 protein expression levels in testes of control and flutamide-treated males (**e**). The relative level of ZO-1 protein normalized to β-actin which served as an internal protein loading control. Data obtained from three separate analyses are expressed as mean ± SD. Significant difference from control values is denoted as *p < 0.05. Control (*n* = 6) and flutamide (*n* = 6) group

As shown by immunohistochemistry, in control rats the ZO-1 signal appeared at the entire cell surfaces of Sertoli cells (Fig. 5a, a’), whereas flutamide treatment resulted in the protein delocalization; the ZO-1 staining frequently dispersed in the cell cytoplasm at the base of the epithelium (Fig. 5b, b’). The changes in the staining pattern of ZO-1 were accompanied by reduction in the staining intensity (Fig. 5b, b’*versus *a, a’). Optical density evaluation revealed significant decrease in ZO-1 immunoexpression (*p* < 0.01) between flutamide-treated and control rats (20.23 ± 1.66 *versus* 32.09 ± 2.16), respectively. Although immunoreactivity of ZO-1 at the BTB was easily recognizable a mild non-specific staining across the epithelium was also visible.

Real time RT-PCR and Western blot analyses revealed down-regulation of both ZO-1 mRNA expression (*p* < 0.05) (Fig. 5c-d) and ZO-1 protein level (*p* < 0.05) in flutamide-treated rats when compared with the control (Fig. 5e-f ), respectively.

### Effects of flutamide on testosterone and estradiol concentrations in testicular homogenates

After flutamide exposure testosterone concentration (9.38 ± 2.3 ng/mg tissue) and estradiol concentration (19.0 ± 2.0 pg/mg tissue) significantly increased (*p* < 0.01) compared with the control values (5.43 ± 2.7 ng/mg tissue and 8.0 ± 1.0 pg/mg tissue, respectively), however, testosterone to estradiol ratio significantly decreased (497.13 ± 128.07 in flutamide-treated rats *versus* 759.13 ± 194.25 in control rats; *p* < 0.05).

## Discussion

Although the tight and adherens junction protein expression have been characterized as androgen-dependent [29, 30] few data are available regarding the action of anti-androgens on the gap junction organization and *Cx43* gene expression in adult rat testis [5]. Our results suggest that short, postnatal exposure to flutamide may have a primary impact on the gap junction organization in the testis. This notion is supported by the following observations: (i) flutamide treatment led to the several ultrastructural alterations of the basal ES (including the number of junctions) and changes in the Cx43 expression at the mRNA and protein level, (ii) localization of the ZO-1 and Cx43 were also affected after flutamide exposure, (iii) this treatment did not lead to histological disturbance of the seminiferous epithelium, and (iv) did not cause obvious changes in cell apoptosis and proliferation.

We demonstrated up-regulation of *Cx43* gene expression in testes of flutamide-treated rats with concomitant reduction of Cx43 immunoexpression and loss of the signal from the Sertoli cell membranes at the BTB, although the Cx43 signal was almost unchanged at the Sertoli cell-elongated spermatid interface. The question aroused whether diminished Cx43 expression at the BTB (as a consequence of anti-androgen exposure) may be related to structural changes in the cell-cell junctions seen at the ultrastructural level. As shown by electron microscopy, cell surface occupied by the basal ES connecting adjoined Sertoli cells was apparently reduced, the number of 3-membrane junctions within the basal ES was clearly lowered (indicating a distinct reduction in the number of cell projections interdigitating between adjacent Sertoli cells), and the number of gap and tight junctions coexisting with the basal ES was reduced in flutamide-treated rats compared with the controls. We suggest that the reduction of the surface occupied by the basal ES is a consequence of internalization of ES segments into the Sertoli cell cytoplasm. This hypothesis is strongly supported by the presence of unconventional circular ES close to the Sertoli cell surface observed only in flutamide-exposed rats. Morphologically similar internalization (“endocytosis”) of intercellular (gap) junctions have been earlier reported by Jordan et al. [31] and Leithe et al. [32]. The authors have also shown that internalized gap junctions (termed annular gap junctions) form double-membrane vacuoles that integrate with endosomes and are finally degraded by endolysosomal proteases. We hypothesize that similar mechanism of endosomal degradation operates also during reduction of the basal ES surface in the system presented herein. This issue is currently under study in this laboratory.

Moreover, we showed reduced tight junction-associated protein (ZO-1) expression at the mRNA and protein level after flutamide exposure. Down-regulation of *ZO-1* gene could reflect impaired functionality of the basal epithelium since the ZO-1 delocalization from Sertoli cell membranes to the cell cytoplasm was evident. This idea is strengthened by the electron microscopy results. Due to our fixation procedure the functional state of the basal ES was visualized. In rats treated with flutamide, some junctional complexes (ES and coexisting TJs) were permeable being penetrated by the electron-dense tracer, whereas in control rats the tracer freely penetrated only between non-specialized membranes. Although we do not know what is the reason of such effect, we think that it is caused by relatively mild conditions of the pre-fixation.

Further, it was important to give an answer whether the expression of ZO-1 in the seminiferous epithelium is somehow related to the changes of the Cx43 expression observed in flutamide-treated rats. It was established that Cx43 physically associates with ZO-1 and ZO-1 regulates gap junction organization in cardiomyocytes and Sertoli cell line [33, 34]. Thus, we cannot exclude the possibility that diminished immunoexpression of Cx43 at the BTB site of flutamide-treated rats may result, at least in part, from decreased ZO-1 expression. Indeed, the reduction of ZO-1 signal and its delocalization from the membrane to the cytoplasmic compartment was reported as related to degradation of the Cx43 gap junction [35]. Interestingly, a role of kinase Src in the interaction of Cx43 with ZO-1 and in the internalization of the BTB proteins was demonstrated [33, 36, 37]. In accord, we have recently found that flutamide-induced disassembly of adherens junctions at the BTB is related to altered Src distribution at this region [20]. We suggest, therefore, that in flutamide-exposed rats delocalization of Cx43 and ZO-1 at the basal ES might be associated with changes in Src functioning.

Of note, we did not find any morphological alterations in the seminiferous epithelium and any obvious changes in cell apoptosis and proliferation after flutamide treatment. In agreement, Woolveridge et al. [38] demonstrated that apoptosis and expression of apoptosis-related proteins were unchanged following short-time androgen signaling disruption. Also Okahara et al. [39] provided evidence that in seminiferous tubules of rats treated for 2 weeks with flutamide at a dose 60 mg/kg/bw no degeneration or decrease of germ cells was observed. On the other hand, increased germ cell apoptosis and histological lesions in seminiferous epithelium were detected in several androgen withdrawal and AR inhibition models, in which androgen depletion lasted for relatively long period [40, 41].

Our morphometric analysis, however, demonstrated significant enlargement of the interstitial tissue with concomitant reduction of Leydig cell number per unit area after flutamide exposure. Since cell proliferation and apoptosis were unaffected, this phenomenon could be attributed to Leydig cell hypertrophy rather than hyperplasia. The Leydig cell hypertrophy was also detected in adult boars exposed to flutamide during neonatal period [23]. In contrary, the study by Okahara et al. [39] based only on qualitative morphological examination (H-E staining), reported increased Leydig cell proliferation after flutamide exposure. To evaluate the function of Leydig cells in flutamide-treated rats, testosterone and estradiol concentrations were measured in testes homogenates. Besides increased concentrations of both steroids, possibly associated with interstitial tissue enlargement, we found significant reduction of testosterone : estradiol ratio in flutamide-treated males compared to the controls. The latter observation may indicate enhanced androgen aromatization in testes of experimental animals, and adds to the explanation of morphological changes in the interstitium. Indeed, previous studies showed that excess of testicular estrogen, due to disruption of estrogen sulfotransferase gene, is associated with Leydig cell hypertrophy [42]. On the basis of the latest findings by O’Hara et al. [43] who generated model of Leydig cell AR knockout mice exhibiting Leydig cell hypertrophy and altered steroidogenesis, we propose that structural and functional abnormalities of the interstitial tissue in flutamide-exposed rats may result, at least in part, from impaired autocrine Leydig cell AR signaling.

Interestingly, in the apparently enlarged interstitial tissue, the Cx43 signal was very strong. The abundant distribution of Cx43 immunoreactive protein at the Leydig cell membranes suggests a potential involvement of Cx43 in the steroidogenic function of Leydig cells. It is likely that coordination of secretory activity among neighbouring cells within the interstitial area may be ensured by gap junction functioning, as previously proposed by Meda [44] for endocrine pancreas cells. It should be added that in our studies the Cx43 expression has been detected in homogenates of whole testes, therefore, up-regulation of *Cx43* gene expression in flutamide-treated testes might be explained by a massive accumulation of the protein within enlarged Leydig cell clusters. Moreover, we earlier demonstrated that enhanced Cx43 immunoexpression was accompanied by increased level of aromatase in hypertrophic Leydig cells of Klinefelter patient [45]. Thus, an increase in Cx43 expression level in the Leydig cells of flutamide-treated rats may result from intratesticular steroid hormones’ imbalance. Why androgen withdrawal (after flutamide exposure) induces different effects on the Cx43 protein expression in various cellular targets remains unclear. It seems possible that various seminiferous tubule compartments and the interstitial tissue are not equally sensitive to androgens and diverse signaling pathways are modulated by the same anti-androgen in different cell types. As we have recently demonstrated, Akt kinase-dependent pathway activation is involved in the reduction of Cx43 protein level in rat Sertoli cells *in vitro* exposed to hydroxyflutamide, an active metabolite of flutamide [21].

## Conclusions

The results reported herein are the first data demonstrating that short treatment with flutamide affects the level of Cx43 mRNA and protein expression, but has no effect on testicular cell proliferation, apoptosis, and the seminiferous epithelium histology. Altered distribution of Cx43 and ZO-1 at the BTB region and altered expression of both proteins suggest that coexisting junctional complexes and their proteins are primary targets for flutamide action. We show also that flutamide exerts its primary effect on the morphology of the basal ES without any apparent histological alterations of the seminiferous epithelium. On the other hand, flutamide action on Leydig cells is manifested by both structural and functional changes in the interstitial compartment*,* involving altered steroidogenic activity and increased Cx43 expression. These observations suggest diverse mechanisms of flutamide action in different cellular targets within the testis of adult rat.
